# A game of persuasion: influencing persuasive game appraisals through presentation frames and recommendation sources

**DOI:** 10.3389/fpsyg.2023.1173429

**Published:** 2023-09-08

**Authors:** Marloes A. Groen, Ruud S. Jacobs

**Affiliations:** Communication Science Research Group, University of Twente, Enschede, Netherlands

**Keywords:** persuasive games, media selection, framing, hedonic, eudaimonic, recommendations, electronic word-of-mouth

## Abstract

**Introduction:**

As games made with the explicit or implicit purpose of influencing players’ attitudes, persuasive games afford a new way for individuals to reflect and elaborate on real-world issues or topics. While research points to effects of these games on their players, little is known about their practical impact. The current study focuses on the decision-making process that takes place between first hearing about a game and deciding to play it. Three elements in a game’s presentation to potential players were explored: (1) the way it is framed as an entertaining experience, (2) the way it is framed as intending to persuade its players, and (3) whether it comes recommended by automated systems or through electronic word-of-mouth. These factors were chosen in line with theoretical arguments around framing, eudaimonia, and source credibility.

**Methods:**

A two (entertainment frame: hedonic versus eudaimonic) by two (persuasive intent frame: obfuscated versus explicit) by two (source of recommendation: system- versus peer-based) between-subjects experimental design was performed across (*N* = 310) randomly distributed participants. Measures were adapted from previous research and included selection and play behavior, attitudes, and obtrusiveness of persuasive intent, among others.

**Results and Discussion:**

Results show that frames need to be congruent to be effective, with the most effective stimuli being those where persuasive intent was clear and players could expect to engage meaningfully. Peer recommendations led to greater play intention than system-based varieties. While intention to play positively related to actual play behavior, this relationship was likely the result of avid game players displaying more interest in the game regardless of the study’s manipulations. Implications are drawn from the advantages of being open about persuasive intent and the composition and drivers of a persuasive game’s target audience.

## 1. Introduction

Despite colloquial perceptions, not all games have the sole purpose to entertain players. The concept of persuasive games can be used to identify a subset of (digital) interactive experiences that are made with the explicit or implicit intent of influencing players’ attitudes toward real-world issues and topics. In some cases, this influence is meant to nudge or steer behaviors without players’ explicit awareness of any influence (Kaufman et al., 2021). In other instances, it might be desirable to invite players to reflect and elaborate consciously on the topic at hand. One game that fits in the latter category is ‘Why did the chicken cross the road?’(WDtCCtR, DatJuanDesigner, 2020). Published on Itch.io by a developer with the username DatJuanDesigner, WDtCCtR is part of an unexplored wave of persuasive games that are emerging on highly accessible game platforms. The game takes a classic set-up for a frivolous joke and uses it to immerse players in the discourse surrounding migrant workers who cross the Mexico-United States border. Even though it advertises itself as a ‘poem/game’ and couches its messaging in unassuming visuals and obfuscatory character dialog, this interactive experience clearly intends for its players to think more critically about this phenomenon. The current study investigates which of these different persuasive messaging styles makes the game most attractive to potential players.

Persuasive games have been studied mostly for the effects they have on players’ attitudes. Some exceptions notwithstanding, this avenue of research generally points to small but robust persuasive influences on diverse social, political, and advertising topics (Jacobs and Jansz, 2021). Crucially, all effects research on persuasive games published so far has included study participants as captive audiences for these games. The tentative conclusion the aggregate of these studies has yielded is that persuasive games tend to persuade the people that play them. This conclusion on effects does not say much about persuasive games’ real-world impact, though. Only a few persuasive games gain public attention. As technological advancements and platforms such as Itch.io are lowering the barriers for independent game developers like DatJuanDesigner to bring their creative ideas to fruition, this is not likely a question of supply. This leaves the processes of discovery, attraction, and selection as potential bottlenecks for the impact that persuasive games can have.

When placed alongside each other, persuasive games appear much the same as entertainment games. They can have colorful visuals, attractive gameplay elements, and interesting titles. However, research on serious games tends to center educational experiences and advergames, which are most often integrated either in a curriculum or an intervention, or they are hosted exclusively in places where their creators think they will be most effective. If research on adoption and acceptance of serious gaming in these formal settings is already rather scarce (see Bourgonjon et al., 2010 for a rare discussion), it is practically non-existent in an open setting where potential players have a bevy of choices on how to spend their time. At the time the current study was performed, one of the most prominent sources for these experiences is Itch.io. This is a platform with low barriers to entry for developers, and since developers can make their games available at multiple price points or even entirely for free, it has proven to be a refuge for independent persuasive game developers in a time when games running on Adobe Flash had been rendered inaccessible to most. The current study engages with the different ways developers can use platforms like Itch.io to draw attention toward their games. More specifically, we investigated how the game’s presentation text and placement of persuasive games on independent gaming platforms relate to the chances they are selected and eventually played. Three elements in a game’s presentation to potential players were explored: (1) the way it is framed as an entertaining experience, (2) the way it is framed as intending to persuade its players, and (3) whether it comes recommended by automated systems or through electronic word-of-mouth (eWOM).

The way we define persuasive games relies on intent on the developers’ side. Of course, would-be players do not always have access to information about developer intent. On top of that, developers themselves might not consider their games as being persuasive, opting instead for seeing their games as ‘starting a discussion’ (Breuer and Bente, 2010), or refusing to classify the persuasive aspect altogether by referring to their work as poetry – as with WDtCCtR. We posit that the degree to which developers are forthcoming about their intentions might impact potential players’ assessment of what the game is as well as the interest they would have in playing it. When writing a description of a persuasive game, one aims to positively influence attitudes and increase the chance of selection. Therefore, it might be worthwhile for the description to appeal to what players are looking for in a game.

In line with previous studies investigating individuals’ reasons for entertainment media selection (Tsay-Vogel and Krakowiak, 2016), we distinguish between hedonic and eudaimonic frames that could both drive media consumption, albeit for different reasons. If the goal is to appeal to hedonic motivations, texts should emphasize fun, enjoyment, happiness, and positive experiences (Oliver and Raney, 2011). This is true for many kinds of gaming experiences, but it is not the only way to pique player interest. Though research on the topic is nascent and a recent overview (Daneels et al., 2021a) noted diverging perspectives and operationalizations, early evidence supports the assertion that games are also played because players appreciated a game’s plot and experience relatedness for in-game characters or real people (Kümpel and Unkel, 2017). At least for pro-social works, it is easy to imagine persuasive games appealing to these types of motivations. Attract texts could appeal to eudaimonic motivations by emphasizing experiences that are meaningful, reflective, and engendering a deeper understanding of a real-world issue.

*H1*: Compared to hedonic frames, eudaimonic frames make it more likely that a persuasive game is selected over an entertainment game.

Still, the eudaimonic rewards persuasive games might have for players are epiphenomenal to games’ intent. Given the rise of entertainment games that include or focus on eudaimonic gratifications (Daneels et al., 2021b), persuasive games stand out from their peers by (typically) being very clear about persuasive intent. The real-world topic they are concerned with might initially be packaged in euphemisms to avoid aversive reactions (Crecente, 2014), but these games often wear the intended influence on their sleeve. Kaufman and Flanagan (2015) recommend a blend of explicit and ‘embedded’ design elements, the latter of which obfuscate the game’s intent to avoid psychological reactance (Brehm and Brehm, 1981) in players who recognize the persuasive attempt. Reactance can cause frustration in players, resulting in rejection of the message as well as the game itself. Of course, reactance is not a given outcome of increased persuasion knowledge (Friestad and Wright, 1994). Some tentative conclusions on persuasion knowledge in persuasive games even posit it as a positive; persuasive games are liked more when their persuasive intent is clearer to players (Jacobs, 2017, 8). The question is still open whether these games should advertise their intent beforehand.

*H2*: Compared to obfuscated persuasive intent, explicit persuasive intent makes it more likely that a persuasive game is selected over an entertainment game.

In the current study, the entertainment frame was manipulated independently from the persuasive intent frame. Of course, their effects might overlap. Eudaimonic motivations center on meaning, reflection, and growth, while explicit persuasive intent (of a pro-social game) simply highlight that the game is intended to have players reflect or take the perspectives of others. These elements might be congruent, potentially leading to additive or amplified effects (Malliet and Martens, 2010). As there has not been any evidence to build on in this regard, we formulated the following research question:

*RQ1*: Do entertainment and persuasive frames interact in their effects on the likelihood that a persuasive game is selected over an entertainment game?

Selection behaviors are not the only measures that could provide insight into the process from discovery to play behaviors. The current study included an interest measure to give depth to the dichotomous selection variable. Participants were also asked about their attitudes toward the game, whether they intended to play the game and, in a one-week follow-up survey, whether they actually sought out the game. Hypotheses were drawn up for the first three of these measures, following the line of argument of H1 and H2 and RQ1.

*H3*: Compared to hedonic frames, eudaimonic frames lead to (a) greater interest to play, (b) more positive attitudes toward, and (c) more intention to play a persuasive game.

*H4*: Compared to obfuscated persuasive intent, explicit persuasive intent leads to (a) greater interest to play, (b) more positive attitudes toward, and (c) more intention to play a persuasive game.

*RQ2*: Do entertainment and persuasive intent frames interact in their effects on (a) interest to play, (b) attitudes toward, and (c) intention to play a persuasive game?

Furthermore, the mechanism theorized for the influence of explicit persuasive intent leans on individuals’ conscious awareness of this intent (Mallinckrodt and Mizerski, 2007). Persuasion knowledge would be the primary mechanism for any effects of this variable, leading to the following hypothesis:

*H5*: Perceived obtrusiveness mediates the relationship between persuasive intent frame and (a) likelihood of selection of, (b) interest in, and (c) attitude toward a persuasive game.

Looking beyond the elements a developer can affect directly, the current study also observed the source of recommendation as a potentially strong influence on the discovery process. Given the tendency for contemporary persuasive game developers to publish their work on independent game platforms like Itch.io, two particular recommendation sources could be salient. Online recommendations can be categorized into system- or consumer-generated varieties (Ashraf et al., 2018). System-based recommendations are automated messages from the platform, typically involving personalized elements by using aggregated user data to support the recommendation. Consumer-generated recommendations could consist of reviews or testimonials by strangers (Lin, 2014), though they are seen as most effective when they come from a peer or familiar person (Kudeshia and Kumar, 2017). Because of the personal nature of a peer-based recommendation, we expected stronger intentions to play following this type of recommendation than a system-based version. The difference is expected to be the result of the peer-based recommendation being perceived as more credible than the system-based recommendation (Luo et al., 2013).

*H6*: Compared to a system-based recommendation, a peer-based recommendation for a persuasive game results in higher intention to play this game.

*H7*: Recommendation source credibility mediates the relationship between recommendation source and intention to play.

The present study was designed to also give insight into whether the proximal measurement of intention to play ultimately translated into (self-reported) play behavior. Extensive prior research and theorizing on the topic demonstrated a relatively robust link between intentions and behaviors (Ajzen, 1991).

*H8*: Intention to play a persuasive game is positively related to subsequent play behavior.

**Figure 1:**
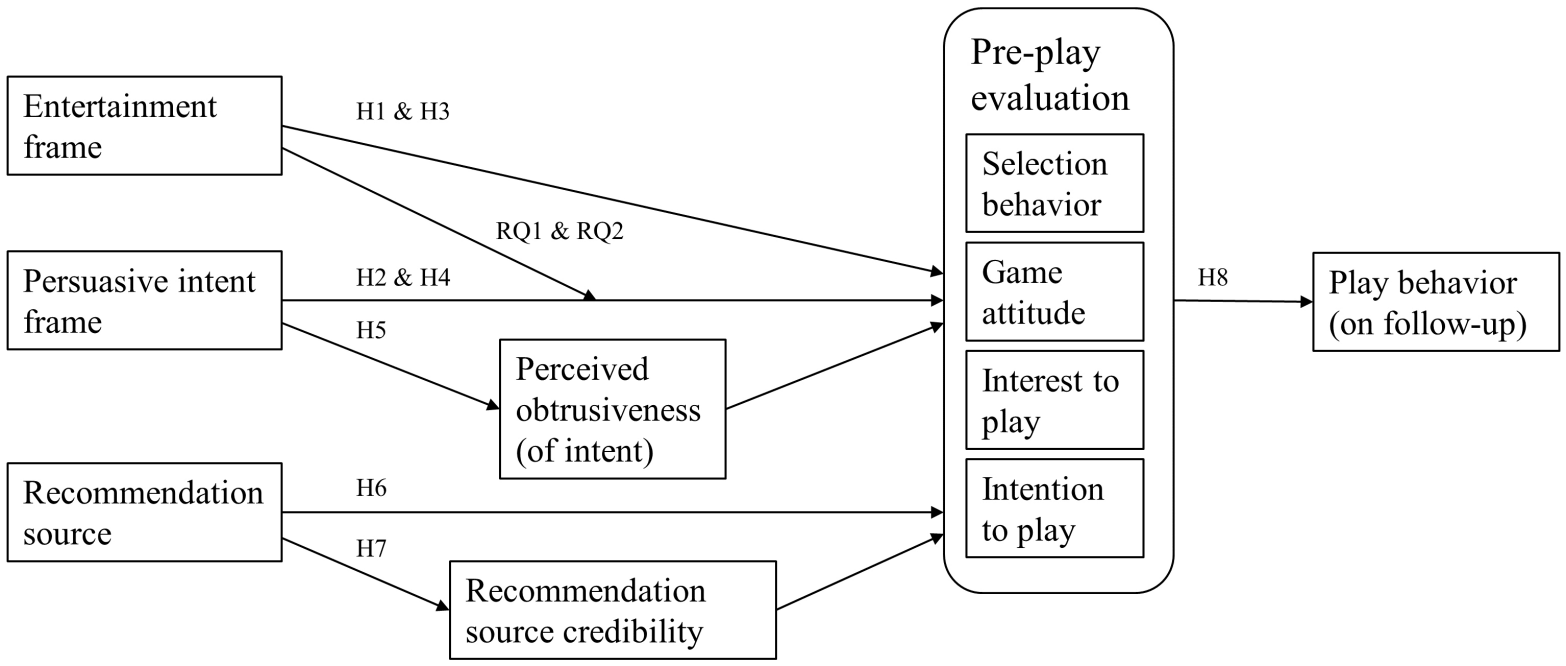
Conceptual model of the influences of presentation frames and recommendation sources on pre-play evaluations and subsequent play behavior. Pre-play evaluation variables are encapsulated to improve readability.

The relationships investigated in this study are displayed in a conceptual model in Figure 1.

## 2. Materials and methods

### 2.1. Design

A two (hedonic or eudaimonic entertainment frame) by two (obfuscated or explicit persuasive intent) by two (system- or peer-based recommendation) between-subjects online experiment was performed. The game ‘Why did the chicken cross the road’ by DatJuanDesigner was selected as the target persuasive game. Participants were exposed to stimuli in two stages to guide them through a facsimile of the process from discovery to persuasive game selection. Stimuli were mock versions of game platform pages and social media application screens. Measurements were taken directly after exposure to a manipulated game presentation text and again after exposure to a manipulated recommendation. Additionally, a follow-up survey was sent to respondents after 1 week that included measures on interest in and experiences with the game. The study’s design was reviewed by the University of Twente’s BMS Ethical Review board and approved for data collection on April 22nd, 2022 (reference 220404).

### 2.2. Participants

The sample for the current study was recruited through convenience sampling among students at the University of Twente in the Netherlands and the principal investigator’s social media reach. This sampling strategy was not expected to yield strong involvement with the game’s topic (the US-Mexican border). A total of 370 respondents met the inclusion criteria of being at least 18 years old. After removing incomplete responses (49) and respondents who did not stay on the stimulus pages for at least 20 s (11), a full sample of (*N* = 310) participants were included for data analysis. Of the full sample, 206 (66.5%) were women, and 101 (32.6%) were men. The average age was 36.20 years (*SD* = 14.40), with a mode of 25 (*N* = 22). Most respondents had either a considerable amount of experience playing games (129, 41.6%) or at least some (70, 22.6%), with 111 respondents (35.8%) claiming little to no experience with the medium. Finally, almost all respondents were European (266, 85.8%), but there were also respondents from the United States of America (37, 11.9%) that might have deviated from the rest of the sample in terms of their involvement with the issue. The distribution of the sample across conditions is shown in Table 1.

**Table 1:** Distribution of respondents across conditions.

	Entertainment frame: *Hedonic*		Entertainment frame: *Eudaimonic*	
	Persuasive intent frame: *obfuscated*	Persuasive intent frame: *explicit*	Persuasive intent frame: *obfuscated*	Persuasive intent frame: *explicit*
Source of recommendation: *System-based*	*N* = 39 71.8% female	*N* = 35 80.0% female	*N* = 39 64.1% female	*N* = 42 66.7% female
Source of recommendation: *Peer-based*	*N* = 43 69.0% female	*N* = 39 57.9% female	*N* = 35 48.6% female	*N* = 38 78.4% female

### 2.3. Stimuli

The game ‘Why did the chicken cross the road?’ (WDtCCtR) was chosen as the target persuasive game because of (among others): its free availability, online accessibility, and short play time which minimized barriers to play; its innocuous metaphor-based visual theming which forces potential players to rely on textual cues to get a sense of the game; its messaging as a pro-social persuasive game; and its relative obscurity helping to prevent familiarity among respondents. The game’s messaging regards the motivations of migrant workers and refugees to cross the Mexico-US border without going through customs. The game demonstrates the hardships these groups of people face through colloquial phrases and anecdotes often voiced by these groups and opponents of this practice in the US. WDtCCtR encourages players to gather more information through charity organizations as well as to donate to help migrant workers and refugees.

#### 2.3.1. Framing presentation text

Four different descriptions of WDtCCtR were composed, differentiated by their entertainment and persuasive intent frames. Table 2 shows the presentation texts with manipulated elements highlighted. The texts included a basic description that was constant across conditions. In line with common distinctions between hedonic and eudaimonic experiences, entertainment frames focus on presenting the game as either a distracting, enjoyable experience or as a meaningful, insightful experience that challenges players’ worldviews. Independently, the game’s persuasive intent was either obfuscated, by presenting it as an adventure game about work and life struggles that raises awareness (without referring to its topic concretely), or made explicit by describing it as a persuasive game that aims to increase sympathy for migrant workers and refugees. In keeping with their positioning as a blurb or attract text, the texts ranged in size from 59 to 69 words across the four versions. The texts were shown together with WDtCCtR’s title screen, which displayed the titular question above a pixel drawing of a yellow chicken standing on a gray road with a yellow background.

**Table 2:** Game presentation text manipulations.

		Persuasive intent frame	
		*Obfuscated*	*Explicit*
Entertainment Frame	*Hedonic*	Escape daily life for a bit, playing *Why did the chicken cross the road?* You must talk to other animals to find the answer to that question. This five-minute **adventure game** offers an enjoyable experience about **work and life struggles**. It aims to **raise awareness** for these struggles. It has a storyline that makes you want to continue playing; you will have fun!	Escape daily life for a bit, playing *Why did the chicken cross the road?* You must talk to other animals to find the answer to that question. This five-minute **persuasive game** offers an enjoyable experience about **work and life struggles of migrant workers and refugees**. It aims to **increase your sympathy** for these struggles. It has a storyline that makes you want to continue playing; you will have fun!
	*Eudaimonic*	Gain some insight, playing *Why did the chicken cross the road?* You must talk to other animals to find the answer to that question. This five-minute **adventure game** offers a meaningful experience about **work and life struggles**. It aims to **raise awareness** for these struggles. It has a touching storyline; your way of viewing the world will be challenged!	Gain some insight, playing *Why did the chicken cross the road?* You must talk to other animals to find the answer to that question. This five-minute **persuasive game** offers a meaningful experience about **work and life struggles of migrant workers and refugees**. It aims to **increase your sympathy** for these struggles. It has a touching storyline; your way of viewing the world will be challenged!

Entertainment frame elements are underlined and persuasive intent frame elements are in bold.

#### 2.3.2. Recommendations

**Figure 2:**
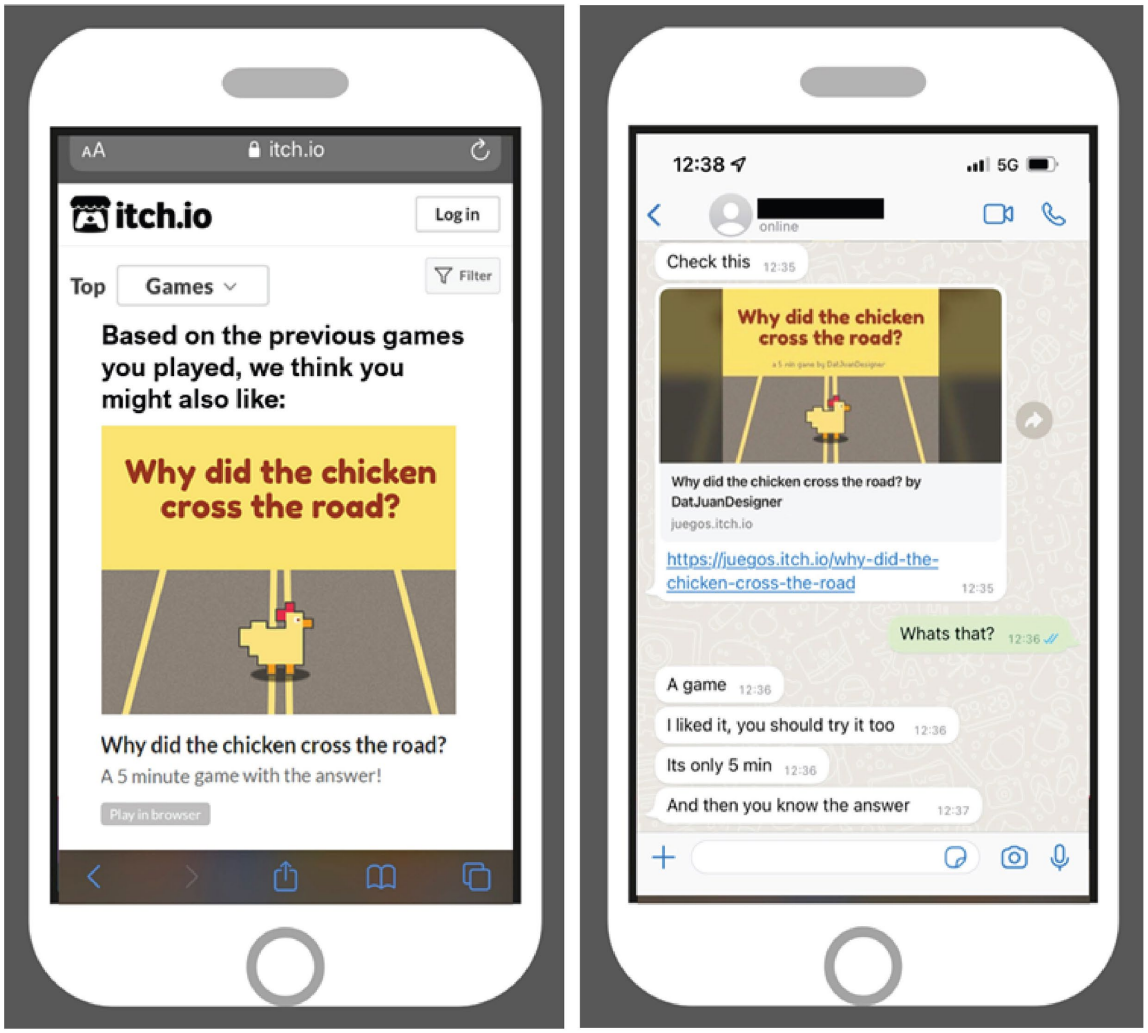
Recommendation source stimuli. Left: system-generated recommendation. Right: peer-based recommendation. Game images (DatJuanDesigner, 2020) and itch.io logo reproduced with permission of DatJuanDesigner and itch.io.

Next, to the frames, the current study also investigated recommendation sources as a potential influence in the persuasive game discovery and selection process. Two types of recommendations were created; one representing the automatic recommender systems integrated into contemporary media and online shopping platforms, and one representing eWOM through a peer recommending the game via social media. Both stimuli (see Figure 2) were presented on a mock-up phone screen with a short introduction text to brief respondents. In the system-generated recommendation conditions, participants were asked to imagine encountering a recommendation after playing a few games on Itch.io. The recommendation was formatted to look like an Itch.io page and read “Based on the previous games you played, we think you might also like” followed by the WDtCCtR splash page and the text “A 5 min game with the answer!” (referring to the question in the game’s title). The peer-based recommendation was preceded by a briefing message asking participants to imagine getting messaged by a friend on WhatsApp. The stimulus itself took the form of a screen from a conversation on this platform. The messages started with “Check this,” followed by a link to WDtCCtR. The account holder is seen responding with “Whats that?.” The friend’s messages then read “A game. I liked it, you should try it too. Its only 5 min. And then you know the answer.” Neither stimulus was interactive.

### 2.4. Procedure

Data collection took place online between May 19th, 2022 and June 12th, 2022. Participants accessed the Qualtrics survey via a direct link. The Qualtrics survey was optimized for computer and smartphone users. After informed consent, participants were asked for demographic information, including experience and affinity with gaming. Participants were then randomly assigned to one of four presentation text conditions. They were asked to evaluate three pairs of games and choose which game they would prefer to play within each pair. Each pair consisted of an identical background image with different presentation texts and titles per game. The first and last pair shown were distractors. The second pair showed WDtCCtR next to a fictitious game called “Back and Forth” (BaF), with both showing the same image of a chicken standing on a road. BaF’s presentation text was identical across conditions: “Back and forth is a game made for your entertainment. You have to get to the other side of a dangerous road and back again as often as you can. With each crossing, you can gain points. But watch out! You have to do so without getting hit by one of the many cars and losing a valuable life. With easy controls, you can play for as long as you like.” Participants rated their interest in playing each of the six games separately.

Following on from the pairs, participants were informed they would be asked specific questions about one of the games, which was invariably WDtCCtR. They then filled in scales of measures relating to attitudes toward the game and the perceived obtrusiveness of the game’s persuasive intent. Directly afterwards, participants were again randomly assigned to one of the two recommendation conditions. They were shown a briefing and one of the mock-ups of the game (system-based) or social media (peer-based) platform. Finally, participants filled in scales relating to intention to play the game and recommendation source credibility and responded to manipulation check questions asking if the presentation made the game seem like ‘simple fun’ or to ‘make me think’ as well as asking if the text made the game seem like it was designed ‘to influence me’. The recommendation source manipulation checks included asking who recommended the game and how realistic the recommendation was to respondents.

Since WDtCCtR is a freely available, real game, we decided to include a second stage in the study. Directly after finishing the survey, respondents were informed where and how to find the game. Around a day after participating, participants were sent a message reminding them of WDtCCtR. This message also included a link to the game. None of these messages included any more information on the game, nor did they encourage participants to try the game. One week after participation, participants were approached to fill in a short follow-up survey. This brief survey only included questions and scales about behaviors regarding the game and scales focusing on attitudes toward the topic of migrant workers and refugees.

### 2.5. Measurement

Responses were collected for seven scales, staggered across four stages during the study protocol. Before stimuli were shown, participants filled in a scale relating to attitudes toward gaming. Scales on attitude to WDtCCtR and perceived obtrusiveness of the game’s messaging were shown directly after the presentation text stimuli. Following the recommendation stimuli, participants could respond to scales on intention to play and recommendation source credibility. In the follow-up survey, scales revolved around attitudes toward migrant workers and refugees and willingness to help this group. All scales were designed in English but were also available in Dutch. After data collection, all scales were included in one confirmatory factor analysis and subjected to individual reliability analyses. The measures below are the result of these analyses. Unless otherwise specified, scale response options were 7-point Likert scales.

#### 2.5.1. Attitude toward gaming

Five polar items were used to have players indicate whether they found playing games to be undesirable/desirable, not enjoyable/enjoyable, negative/positive, boring/interesting, and harmful/beneficial. These items were adapted from Wang and Hollett’s (2022) scale on physical activity. The scale’s scores demonstrated high internal consistency (Cronbach’s *α* = 0.94).

#### 2.5.2. Attitude toward the persuasive game

The attitude toward the persuasive game included after exposure to the game pairs was measured using a scale adapted from Vanwesenbeeck et al. (2017). Participants were asked to indicate on a seven-point semantic differential scale how they felt about the game, based on the description they had read. The adjectives used were unappealing/appealing, unpleasant/pleasant, dull/dynamic, unattractive/attractive and not enjoyable/enjoyable. This scale’s scores demonstrated high internal consistency (Cronbach’s *α* = 0.94).

#### 2.5.3. Perceived obtrusiveness

Perceived obtrusiveness was a five-item scale adapted from Jacobs (2017) and from Tutaj and Van Reijmersdal (2012). Changes were made to account for the prospective nature of the assessment. An example item is “The aim of the game [WDtCCtR] is to influence my opinion.” This scale’s scores were internally consistent (Cronbach’s *α* = 0.82).

#### 2.5.4. Intention to play

Four items from Spears and Singh (2004) were adapted to the current context. This polar item scale asked participants whether they probably would not play/probably would play, have low/high interest in playing, definitely do not intend to play/definitely do intend to play and would definitely not play/would definitely play. This scale’s scores demonstrated high internal consistency (Cronbach’s *α* = 0.97).

#### 2.5.5. Recommendation source credibility

This five-item polar scale included four items from Amelina and Zhy (2016) and one item from Filieri et al. (2015). Participants were asked to indicate to what extent they thought the source giving them the recommendation was not at all trustworthy/very trustworthy, not at all reliable/very reliable, not at all sincere/very sincere, not at all honest/very honest, and not at all credible/very credible. This scale’s scores demonstrated high internal consistency (Cronbach’s *α* = 0.97).

#### 2.5.6. Attitudes toward migrant workers and refugees

One week after being exposed to the manipulated messaging about the game, a scale focusing on attitudes toward migrant workers and refugees was shown to participants. Four items were adapted from Igartua et al. (2019) and McConahay et al. (1981) with some changes to fit the current context. An example item from this scale was “Migrant workers and refugees are getting too demanding in their push for better treatment.” The scale’s scores showed sufficient internal consistency (Cronbach’s *α* = 0.79).

#### 2.5.7. Willingness to help

The final scale was a three-item willingness-to-help scale. Following van’t Riet et al. (2018) participants could indicate how likely they were to perform three specific behaviors. These behaviors were (1) to donate money to a charity helping refugees and migrant workers, (2) to discuss the situation of refugees and migrant workers with their friends or family, and (3) to do volunteer work involving refugees and migrant workers. This scale’s scores showed sufficient internal consistency (Cronbach’s *α* = 0.77).

#### 2.5.8. Other measures

Directly below each pair of games, participants were asked which of the two games they would prefer to play. This single binary item was only analyzed for the second pair, which included the manipulated texts. Within the sample, 141 participants (45.5%) indicated wanting to play WDtCCtR over the fictitious entertainment game. Interest to play was measured separately with a single item for each of the six games. Responses were given on a scale from 0 (not at all interested) to 100 (very interested). Across all conditions, respondents were not very interested in playing the persuasive game (*M* = 36.20, *SD* = 27.04).

In the follow-up survey, participants were asked three consecutive questions about their behavior with WDtCCtR with yes/no response options. The first was whether they tried to access the game after the study ended. A total of 52 participants (16.8%) had done so. Of those, 40 (12.9% of the full sample) had started playing, and 26 (8.4%) had finished the game entirely.

### 2.6. Data analysis

All analyses were performed with IBM SPSS Statistics v26 and, where applicable, the PROCESS procedure for SPSS v4.2 (Hayes, 2018). Hypothesis and research question testing involved binary logistic regressions (H1, H2, H8, RQ1) Multivariate ANOVAs (H3, H4, RQ2), model 4 of the PROCESS macro (H5, H7), and a univariate ANOVA (H6). Additional analyses employed the same procedures, though linear regression analyses and independent-samples *t*-tests were also performed. The dataset and syntax used for the full set of analyses are available online.^1^

## 3. Results

### 3.1. Hypothesis testing

#### 3.1.1. Framing entertainment and persuasive intent

A logistic regression was performed to test the first and second hypotheses, which stated that eudaimonic entertainment and explicit persuasive intent frames would lead to greater likelihood that a persuasive game is selected over an entertainment game, as well as the first research question, which involved an interaction between both independent variables. Across all conditions, 45.5% of participants selected WDtCCtR. In the condition where a eudaimonic and an explicit persuasive intent frame were combined, 54.8% of participants chose the persuasive game. A model that included both manipulated frame variables and an interaction term as predictors and selection likelihood as the dependent variable was not significant [*X*^2^ (3, *N* = 310) = 3.36, *p* = 0.340]. Neither entertainment frame nor persuasive intent helped to predict when respondents would choose WDtCCtR over its entertainment counterpart. Hypotheses 1 and 2 are both rejected following this outcome. The answer to RQ1 is that there is no evidence for an interaction between both types of frame on the likelihood that the persuasive game is selected.

**Figure 3:**
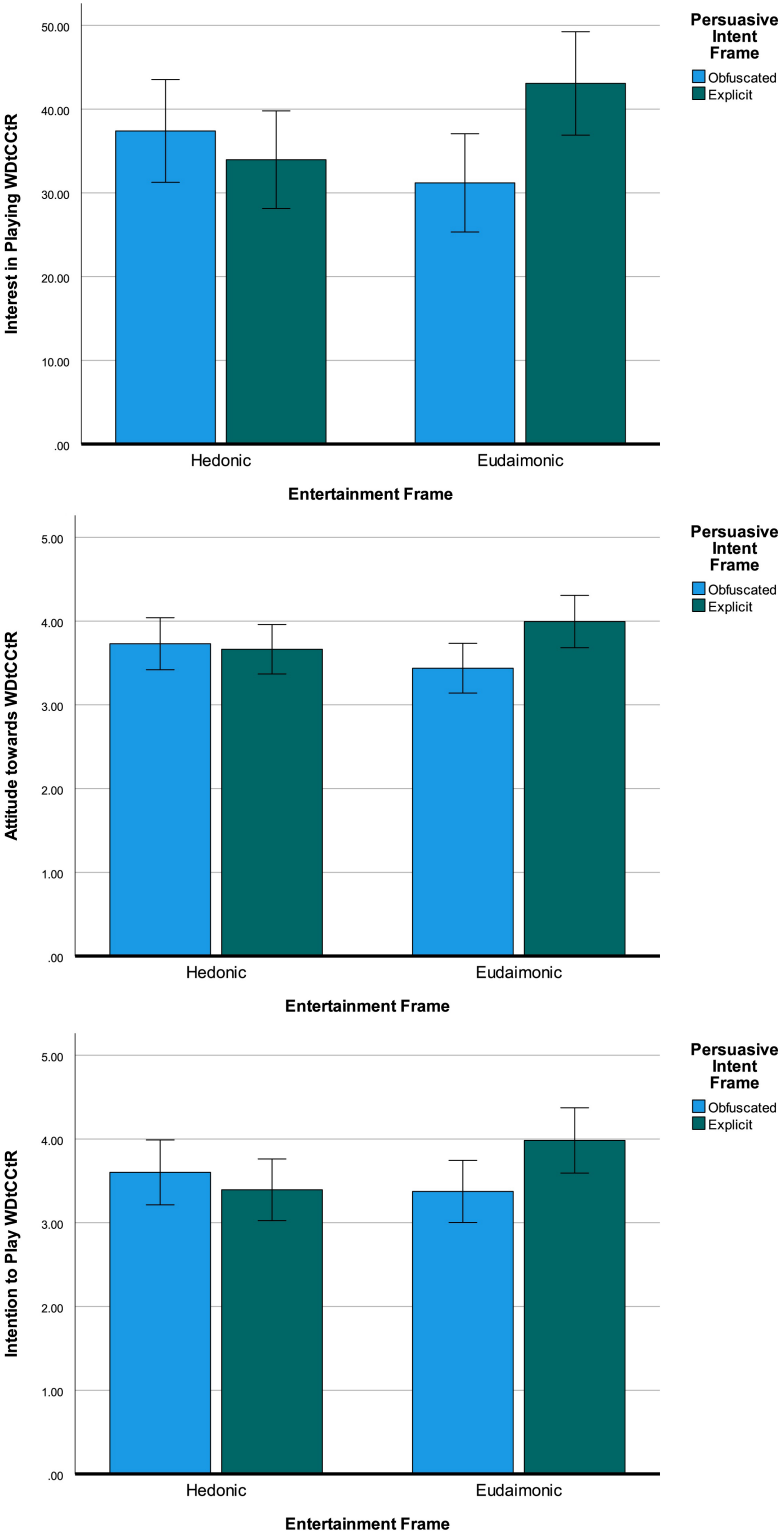
Graphs showing the interaction effect of entertainment and persuasive intent frames on interest in playing the game (top), attitude toward the game (middle), and intention to play the game (bottom). Error bars note 95% confidence intervals.

Hypotheses 3 and 4 and research question 2 centered on the influences that entertainment and persuasive intent frames have on the interest in, attitude toward, and intention to play a persuasive game. A multivariate analysis of variance (MANOVA) was performed with these outcome variables and both main effects and the interaction term of the two manipulated variables. Across all three dependent variables, both main effects were not significant [Entertainment frame: *F*(3, 304) = 0.37, *p* = 0.772, Wilk’s Λ = 1.00, Persuasive intent frame: *F*(3, 304) = 0.90, *p* = 0.442, Wilk’s Λ = 0.99]. The interaction term was also not significant over all dependent variables [*F*(3, 304) = 2.39, *p* = 0.069, Wilk’s Λ = 0.98]. None of the dependent variables were significantly affected by the two main effects, but the interaction effect was significant for all three dependent variables individually (Interest: *F*(1, 306) = 6.29, *p* = 0.020, *η*^2^_p_ = 0.02, Attitude: *F*(1, 306) = 4.08, *p* = 0.013, *η*^2^_p_ = 0.01, Intention: *F*(1, 306) = 4.50, *p* = 0.014, *η*^2^_p_ = 0.01], though effects were very small. Figure 3 shows the direction of the interaction effect. Within hedonic entertainment frame conditions, there are no substantial differences between obfuscated and explicit intent on interest in (*M_obf_* = 37.39, *SD_obf_* = 22.67, *M_exp_* = 33.96, *SD_exp_* = 28.70), attitude toward (*M_obf_* = 3.73, *SD_obf_* = 1.26, *M_exp_* = 3.66, *SD_exp_* = 1.52), or intention to play WDtCCtR (*M_obf_* = 3.60, *SD_obf_* = 1.55, *M_exp_* = 3.39, *SD_exp_* = 1.79). However, for those who saw the game presented with a eudaimonic frame, differences between obfuscated and explicit persuasive intent were more pronounced. Combined with a eudaimonic frame, explicit persuasive intent led to higher interest in (*M_obf_* = 31.20, *SD_obf_* = 26.38, *M_exp_* = 43.07, *SD_exp_* = 28.88), attitude toward (*M_obf_* = 3.44, *SD_obf_* = 1.36, *M_exp_* = 3.99, *SD_exp_* = 1.25), and intention to play the persuasive game (*M_obf_* = 3.37, *SD_obf_* = 1.77, *M_exp_* = 3.98, *SD_exp_* = 1.64) than obfuscated persuasive intent did. Hypotheses 3 and 4 are rejected. The answer to RQ2 is that explicit persuasive intent only led to more positive assessments of the game when combined with a eudaimonic frame.

**Figure 4:**
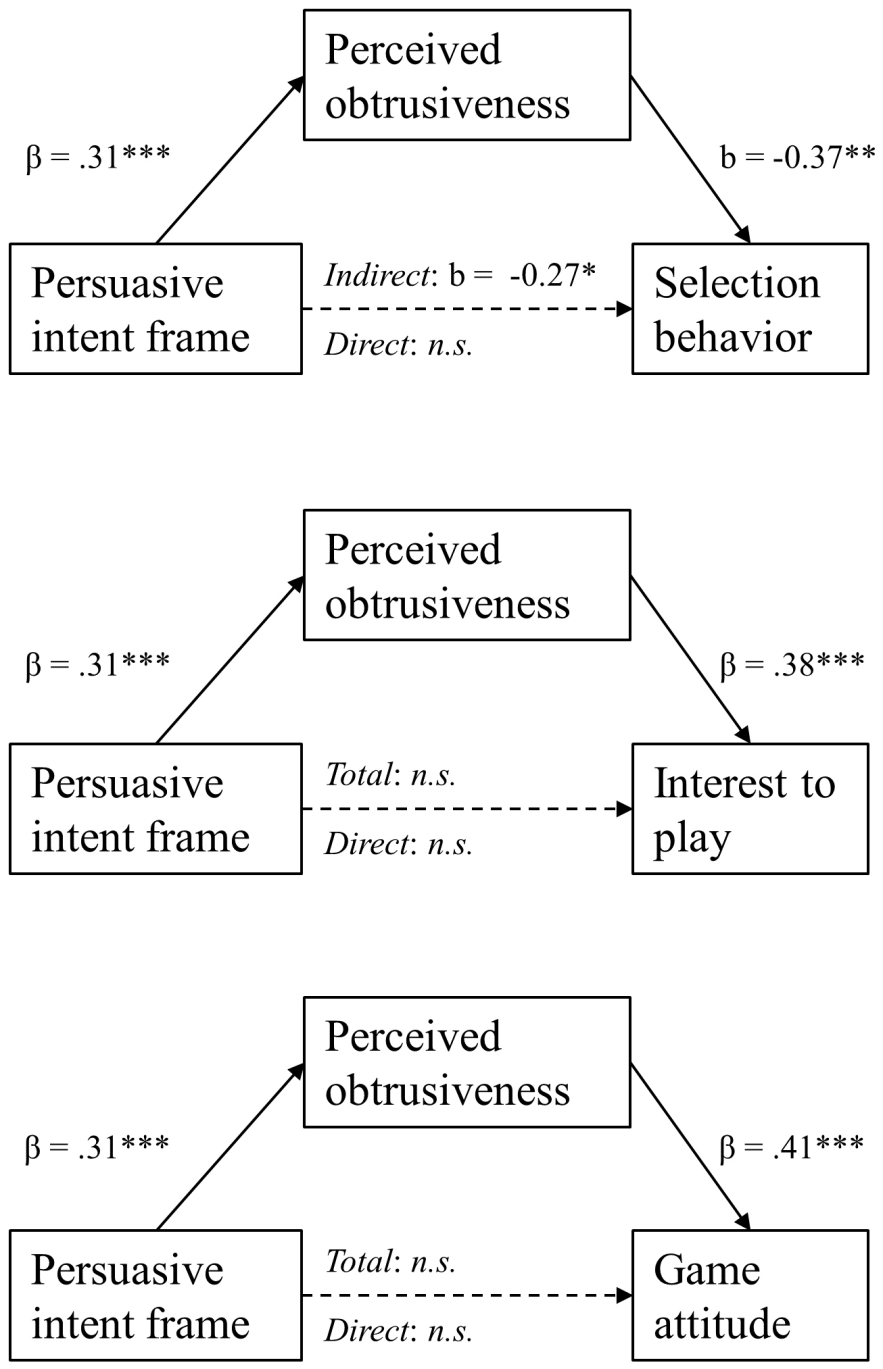
Empirical pathway models on the mediating relationship of perceived obtrusiveness on the effect of persuasive intent frame on selection behavior (top), interest to play the game (middle), and attitude toward the game (bottom).

The fifth hypothesis, which held that the effects of persuasive intent frame on (a) likelihood that the persuasive game is selected, (b) interest in playing this game, and (c) attitudes toward the game are mediated by perceived obtrusiveness, cannot be confirmed given the outcomes for hypotheses 2 and 4. The mediation analyses were still performed to determine whether an indirect effect could be established. Mediation analyses were performed with model 4 of the SPSS PROCESS (Hayes, 2018). An overview of results is shown in Figure 4. Firstly, persuasive intent frame had a significant effect on perceived obtrusiveness [*β* = 0.31, *t*(1, 308) = 5.63, *p* < 0.001]. An obfuscated persuasive intent frame was perceived as less obtrusive (*M* = 3.88, *SD* = 1.13) compared to explicit persuasive intent frame (*M* = 4.60, *SD* = 1.10).

Starting with H5a, a negative relationship between perceived obtrusiveness and selection behavior (a dichotomous variable) was found to be significant [*b* = −0.37, *SE b* = 0.11, Z (2) = −3.40, *p* = 0.007]. To illustrate: participants who chose *WDtCCtR* tended to find this game’s persuasive intent significantly more obtrusive (*M* = 4.50, *SD* = 1.11) compared to those who chose the entertainment game (*M* = 4.02, *SD* = 1.18). The model as a whole explained between 4.2% (Cox and Snell R-square) and 5.6% of the variance in selection behavior. The indirect effect of persuasive intent frame on selection behavior was significant (*indirect effect* = −0.27, *SE* = 0.09, 95% CI [−0.47, −0.12]). As the direct effect of persuasive intent frame on selection behavior was insignificant (*direct effect* = 0.02 *SE* = 0.24, 95% CI [−0.45, 0.50]), there was no evidence to say perceived obtrusiveness mediated this (non-significant) relationship.

Second, we investigated if perceived obtrusiveness mediated the effect of persuasive intent frame on interest to play. Perceived obtrusiveness yielded a moderate, positive, significant relationship with interest to play [*β* = 0.38, *t*(309) = 6.76, *p* < 0.001]. The indirect effect of persuasive intent frame on interest to play was significant (*partially standardized indirect effect* = 0.23, *SE* = 0.05, 95% CI [0.13, 0.34]). Yet both the total effect [*β* = 0.15, *t*(309) = 1.33, *p* = 0.183] and the direct effect [*β* = −0.08, *t*(309) = −0.70, *p* = 0.483] were insignificant, meaning that there was no evidence for mediation occurring. Looking finally at H5c, perceived obtrusiveness was moderately, positively, and significantly related to attitude [*β* = 0.41, *t*(309) = 7.47, *p* < 0.001]. When looking at the total effect of the persuasive intent frame on attitude toward the persuasive game, this was not significant [*β* = 0.18, *t*(309) = 1.57, *p* = 0.118]. Also, the direct effect of persuasive intent frame on attitude was insignificant [*β* = −0.07, *t*(309) = −0.66, *p* = 0.511]. Although the indirect effect of persuasive intent frame on attitude was significant (*partially standardized indirect effect* = 0.25, *SE* = 0.06, 95% CI [0.15, 0.37]), though obtrusiveness was not found to mediate the relationship between persuasive intent frame and attitude toward the game.

#### 3.1.2. Recommendation source

Hypothesis 6 assumed that a peer-based recommendation would lead to higher intention to play compared to a system-generated recommendation. An ANOVA was performed with recommendation source as the independent variable and intention to play as the dependent variable. The model was significant [*F*(1, 308) = 22.30, *p* < 0.001, *η*^2^_p_ = 0.07], with a moderate effect size. Peer-based recommendations led to significantly higher intention to play (*M* = 4.04, *SD* = 1.73) than system-based ones (*M* = 3.15, *SD* = 1.57). As a result, hypothesis 6 is retained.

**Figure 5:**
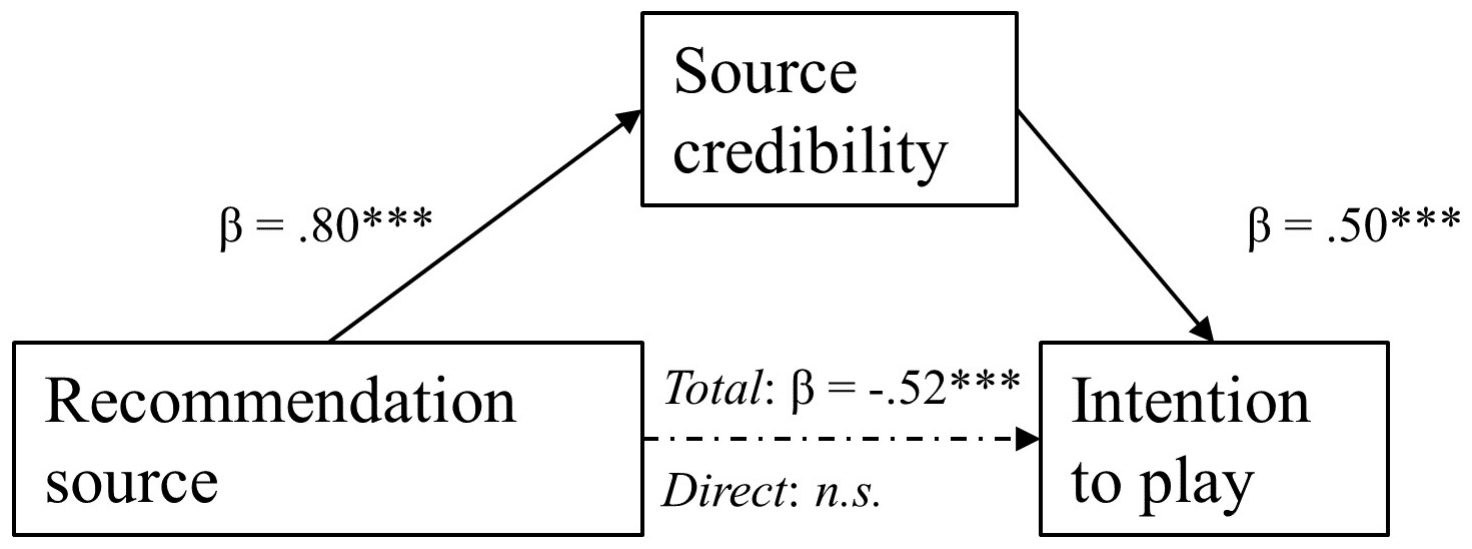
Empirical pathway models on the mediating relationship of source credibility on the effect of recommendation source on intention to play.

To test hypothesis 7, which was concerned with the effect of source of recommendation on playing intentions being mediated by perceived source credibility, Model 4 of PROCESS by Hayes was once more adopted. An overview of results is shown in Figure 5. Source of recommendation had a significant effect on perceived source credibility [*β* = 0.80, *t*(309) = 9.36, *p* < 0.001]. Peer recommendations led to higher perceived source credibility (*M* = 4.76, *SD* = 1.34) than a system-generated recommendation (*M* = 3.55, *SD* = 1.46). Source credibility in turn was positively and significantly related to intention to play [*β* = 0.50, *t*(309) = 9.36, *p* < 0.001]. When looking at the total effect of source of recommendation on intention to play the persuasive game, this was significant [*β* = −0.52, *t*(309) = −4.72, *p* < 0.001]. Meanwhile, the direct effect of source of recommendation on intention to play was insignificant [*β* = −0.12, *t*(309) = −1.17, *p* = 0.244]. As the indirect effect of source of recommendation on intention to play was significant (*partially standardized indirect effect* = −0.40, *SE* = 0.07, 95% CI [−0.55, −0.27]) there was enough evidence to assume that this relationship was fully mediated by perceived source credibility. Therefore, hypothesis 7 is retained.

#### 3.1.3. Follow-up survey

Closing off the hypothesized relationships, we tested H8, which predicted a positive relationship of intention to play with subsequent play behavior. The variable indicating whether or not people reported trying to find WDtCCtR online was taken as the dependent variable. A logistic regression analysis was performed with intention to play the game as the predictor. This resulted in a significant full model [*X^2^* (1, *N* = 287) = 11.41, *p* < 0.001] that explained between 3.9% (Cox and Snell R Square) and 6.4% (Nagelkerke R Square) of the variance in playing behavior. It correctly classified 81.9% of the cases. Each point increase in intention to play (on a scale from one to seven) led to a 38% increase in odds that one attempted to access *WDtCCtR* (*b* = 0.32, *SE* = 0.10, *p* = 0.001, Exp (b) = 1.38, 95% CI [1.14, 1.67]). Therefore, it can be concluded that intention to play is positively related to subsequent playing behavior and hypothesis 8 is retained.

### 3.2. Additional analyses

In addition to hypothesis tests, four additional avenues were explored within the current study’s dataset. These four avenues were (1) the exploration of how much of the variance in playing intention (measured at the end of the main survey) could be predicted from the manipulated and measured variables included in the study; (2) the exploration of potential relationships between the manipulated variables and source credibility; (3) confirming any connection of the manipulated variables with self-reported play behavior in the follow-up survey; and (4) a tentative exploration of how the study’s variables relate to individuals’ attitudes toward the game’s topic (also measured in the follow-up survey).

A linear regression analysis was performed to determine how well the independent variables entertainment frame, persuasive intent frame, perceived obtrusiveness, attitude, interest to play, source of recommendation, and source credibility were able to predict intention to play a persuasive game. The results showed that a model which included these variables explained 52.2% of the variance in intention to play the persuasive game [*F*(7, 302) = 47.11, *p* < 0.001, Adjusted *R*^2^ = 0.51]. Recommendation source, interest to play, attitude, and source credibility were significant predictors in the model. The strongest contribution was made by source credibility (*β* = 0.37, *p* < 0.001), which was a moderate positive predictor of intention to play when controlling for the other included independent variables. Interest to play (*β* = 0.29, *p* < 0.001), attitude (*β* = 0.22, *p* < 0.001), and source of recommendation (*β* = 0.12, *p* = 0.009) were weak positive predictors of intention to play. This final result is surprising, as the mediation analysis performed to find support for H7 pointed to full mediation. As the only difference between this significant relationship and the insignificant direct effect found in that analysis is the inclusion of more independent variables in this last linear regression, explanations need to be sought among the other predictors of intention to play.

**Figure 6:**
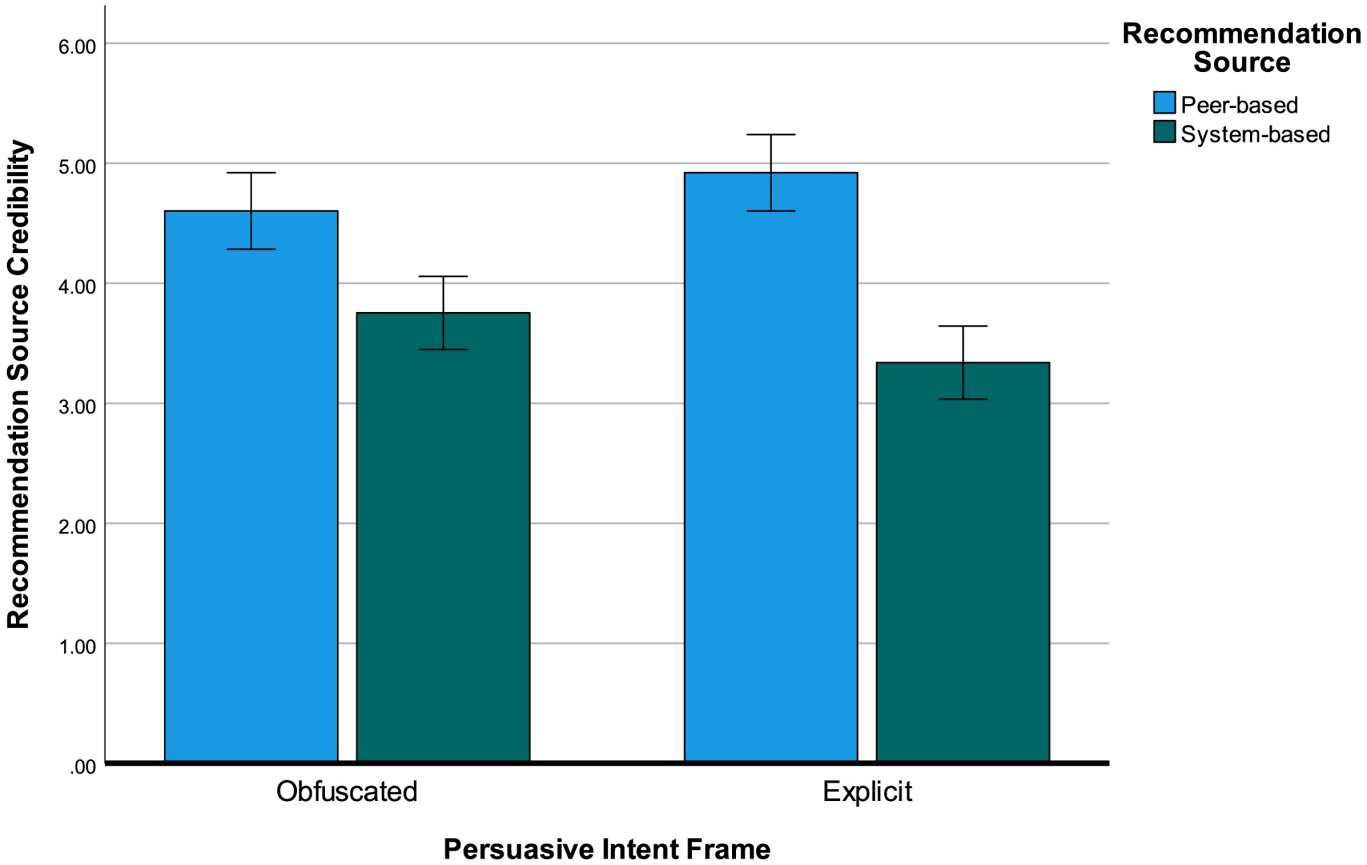
Graph showing the interaction effect of persuasive intent frame and recommendation source on source credibility. Error bars note 95% confidence intervals.

Next, as recommendation source credibility was measured after all manipulated variables had been shown to respondents, there might have been influences on this variable from the two framing manipulations included before. In an ANOVA where both frames and recommendation source were taken into account as main effects and the interaction terms between either frame manipulation and recommendation source were included yielded a significant outcome for recommendation source credibility. While the main effects of both framing variables were not significant [Entertainment: *F*(1, 304) = 2.88, *p* = 0.091, Persuasive intent: *F*(1, 304) = 0.05, *p* = 0.830] and the interaction between entertainment frame and recommendation source was also insignificant [*F*(1, 304) < 0.01, *p* = 0.983], the interaction term between persuasive intent frame and recommendation source was significant [*F*(1, 304) = 5.39, *p* = 0.021, *η*^2^_p_ = 0.02]. This interaction effect is shown in Figure 6. Inspection of mean differences reveals that for both an obfuscated (*M* = 4.60, *SD* = 1.32) and an explicit persuasive intent frame (*M* = 4.92, *SD* = 1.34), a peer-based recommendation led to significant increases in source credibility compared to a system-generated recommendation (*M_obf_* = 3.75, *SD_obf_* = 1.41, *M_exp_* = 3.33, *SD_exp_* = 1.48). The effect of the source of recommendation was amplified when the persuasive intent frame was explicit; the difference between the two types of sources was larger for explicit frames than it was for obfuscated frames. It is important to note that the size of this effect was quite small.

Third, a hierarchical logistic regression was performed to first test for any potential influence of the study’s experimental manipulations on self-reported play behavior. Entertainment and persuasive intent frames as well as recommendation source were included in the first step, with interest to play, attitude toward, and intention to play included together with perceived obtrusiveness, recommendation source credibility, and general attitude toward gaming included in the second step. The first step did not yield a significant model [*X*^2^(3, *N* = 287) = 0.37, *p* = 0.947], meaning that none of the experimental manipulations caused respondents to be more or less likely to have sought out the game within a week after exposure. At step 2, the results showed that the full model was significant [*X*^2^(9, *N* = 287) = 35.33, *p* < 0.001] and thus was able to distinguish between those who did try to access the game and those who did not. As a whole, the model explained between 11.6% (Cox and Snell R Square) and 18.9% (Nagelkerke R Square) of the variance in playing behavior and correctly classified 82.9% of the cases. Attitude toward gaming in general was the only significant predictor of self-reported play behavior (*b* = 0.36, *SE* = 0.14, *p* = 0.008), with an odds-ratio of 1.44 indicating that for every one point (out of 7) one would be more interested in gaming, their likelihood to have sought to play the game would be predicted to have risen by 43.9%. Interest in WDtCCtR itself was not a significant predictor (*b* = 0.02, *SE* = 0.01, *p* = 0.057), though it was closer to the threshold of *p* = 0.05 than any of the other variables. A simple linear regression confirmed that general attitude toward gaming was a moderate predictor of interest in playing WDtCCtR [*F*(1, 308) = 71.88, *p* < 0.001, *β* = 0.44] and intention to play this game [*F*(1, 308) = 28.98, *p* < 0.001, *β* = 0.29]. More avid gamers were more interested in the game.

The final additional analyses engaged with the attitudes people reported holding toward the issue WDtCCtR focuses on. An overall linear regression with the manipulated variables as well as interest to play, attitude toward, intention to play, perceived obtrusiveness, recommendation source credibility and individuals’ attitudes toward gaming in general indicated that these variables could not significantly predict attitudes toward migrant workers and refugees [*F*(9, 277) = 1.23, *p* = 0.277], though it did serve to predict willingness to help [*F*(9, 277) = 2.01, *p* = 0.038]. The only significant predictors in this model were general attitude toward gaming (*β* = −0.17, *p* = 0.012) and interest to play WDtCCtR (*β* = 0.19, *p* = 0.034). General attitude toward gaming was a weakly negative predictor, while interest in the game was a weakly positive predictor of willingness to help migrant workers and refugees. Independent samples t-tests showed that there were no significant differences between those participants who did and those who did not seek out the game in terms of attitudes [*t*(285) = 0.71, *p* = 0.480] or willingness to help [*t*(285) = −0.08, *p* = 0.940].

## 4. Discussion

### 4.1. Discussion of results

The current study investigated the influences of entertainment and persuasive intent frames together with system- and peer-based recommendations on interest in and intention to play a persuasive game. It was clear from the results of this study that entertainment and persuasive intent frames by themselves do not directly affect assessments of this persuasive game. Looking beyond this, frames also do not make it more or less likely that the game is eventually sought out. The pattern of findings beyond this initial result was more intricate, however.

We did find an effect when two specific frames were combined. Being more or less open about the game’s persuasive intent did not seem to matter for participants who were exposed to a hedonic entertainment frame. However, when the text that presented the game emphasized that it would make players think about this topic, interest, attitudes, and intent decreased when persuasive intent was implicit and increased when it was explicit. This effect did not cause participants to select the persuasive game over a similar entertainment game on first exposure, though. These findings could be indicative of a congruence effect (Malliet and Martens, 2010), where texts were seen as consistent if they presented the game as being fun or if they presented the game as eudaimonically gratifying. In the latter case, this would clearly serve to position its persuasive intent as benign; players are invited to reflect on a real-world issue, rather than feeling threatened and pushed to act (conform Staunton et al., 2022). Persuasive intent frames themselves were only indirectly related to selection behaviors and assessments, through obtrusiveness. Explicit persuasive intent frames had an effect on obtrusiveness, which was in turn predictive of interest in the game. The absence of any direct effects in spite of this sizeable indirect link can be interpreted in different ways. While it could be possible that only those who perceived the text as obtrusive (regardless of frame condition) were made to recognize the description as being congruent with a game that aims to persuade as a result of obtrusiveness, the current dataset offers no way to confirm or deny this explanation.

One outcome that was expected was the influence of getting the game personally recommended by a peer compared to a system-generated recommendation. Even within the artifice of the experiment, participants who viewed a peer-based recommendation reported higher play intentions. As current knowledge on the mechanisms of effect of online recommendation systems would suggest (Cheung et al., 2009; Ashraf et al., 2018), this influence was fully mediated by source credibility – at least when no other variables were considered. A small effect from recommendation source on intention to play was found even when source credibility was included only when interest to play and attitude toward the game were also included. These variables preceded exposure to the recommendation stimulus, so including them in the additional linear regression might have made an additive positive effect of the peer recommendation visible next to its mediator, source credibility. Again, since just recommendation source was manipulated and the final effect was quite small, this finding throws up more questions than the current study can answer. What is clear is that for the low-involved participants of the current study, system-generated recommendations are not very persuasive.

The interaction effect between source of recommendation and persuasive intent frames found in the additional analyses provides further evidence for the position that participants were looking for information about WDtCCtR to make their decision whether or not to play it. Having a peer recommend a game that was previously explicitly advertised as persuasive feeds into perceptions of recommendation source credibility. In this and previous findings, entertainment and persuasive intent frames seem to act as cues; they are not necessarily sufficient by themselves to shift attitudes, but are taken into account together with other cues offered simultaneously or subsequently. When these cues are congruent, their aggregate effects give would-be players more certainty in their decisions.

There is a complex set of relationships between individual variables like attitude toward gaming in general and evaluations of the game as well as with self-reported play behavior in the one-week follow-up. Intention to play is likely to be the result of a combination of cues about the game and assessments where those cues are coming from. Looking beyond intention to play, complexity grows even further. When tested separately, intention to play WDtCCtR was positively related to one’s efforts in subsequently putting in effort to play the game. When the full range of variables that was measured during this study were taken into account, it turned out that it was actually the attitudes someone holds toward gaming in general, rather than interest in or intention to play, that predicted the likelihood that someone tried to play this game. A potential explanation for this could be that participants ‘played along’ with the study’s artificial texts and phony recommendations, but that only those participants who already had a prior interest in gaming held onto that interest after the study was over and real life resumed. Support for this explanation comes from attitudes toward gaming positively predicting interest and intention within the study as well. Frequent players were more interested in the game regardless of cues, and it was that contingent of participants who demonstrated affinity with the medium that was more likely to actually try WDtCCtR.

Finally, we found that none of the variables included here could predict players’ attitudes toward migrant workers or refugees. As this was not a study that exposed players to the actual game as part of its manipulations, this finding cannot be taken to mean WDtCCtR is not effective in changing players’ attitudes. It does mean that the cues that were tested here did not evoke more interest among potential players who were already invested in this issue beforehand. The two remarkable findings were that attitudes toward gaming in general and interest in the game itself both predicted willingness to help migrant workers and refugees. The two had opposite effects, though; those with more positive attitudes toward gaming displayed less willingness to help – even though they were more likely to have tried out the game – while interest in the game itself correlated positively with this same variable. Considering the complexity of these relationships and (again) the small effect sizes noted, it is not prudent to connect the negative relationship to a broader pattern of socio-political disengagement among frequent gamers (Bacovsky, 2021), but this finding does highlight that persuasive games’ target audiences and actual player bases still tend to exist in the overlap between those who might be interested in the game itself or the topic, and those who are interested in the medium of gaming regardless of what messages they might hold.

### 4.2. Implications

#### 4.2.1. Theoretical implications

The current study provides tentative evidence in favor of a link between eudaimonic play motivations – a largely unexplored set of motivations favoring meaningful and reflection-inviting content over more straightforward entertainment fare (Daneels et al., 2021a) – and interest in persuasive games. While the strategy to hide persuasive intent through clever design features (Kaufman and Flanagan, 2015) is clearly valid in some respects, the current study suggest that it is not the only viable strategy to get players to engage with persuasive games. Indeed, explicit communication about persuasive intent can make the game more interesting because of this, as players might try to satisfy their curiosity about what the game could be saying about a specific topic. It is an open question whether this is due to the sight of a game (of all things) broaching sensitive socio-political issues representing a novelty for audiences whose prior contact with persuasive gaming was limited to Dumb Ways to Die (Metro Trains Melbourne, 2016), or whether it represents an ongoing shift in the culture and image of gaming whereby players are learning to expect more meaningfulness from their games (Similar to the shift in attitudes toward gaming described by Kneer et al., 2018). The finding that people who have more affinity with gaming also display less willingness to help does not necessarily refute the latter possibility.

Looking more closely at the findings surrounding the game descriptions, it is likely that congruence in messaging plays an important role in players’ ideas about and attitudes toward persuasive games. Congruence is implicated in framing messages causing positive assessments of a persuasive game, as well as with eWOM when it comes to perceptions of obtrusiveness. This makes it somewhat more difficult to advise on further experimentation with frames, as the current study’s messages seem to have been judged holistically. Inconsistent framing elements could dampen effects from manipulations that would be viable in a fully controlled setting.

Finally, the interest that people have in gaming as a hobby is related in complex ways to how people engage with persuasive games. While identities of game players might be broader than ever before across the history of the medium (Howe et al., 2019), they are often still directly tied to being a consumer of the entertainment gaming industry (De Grove et al., 2015). If an experience strays too far from the fuzzy boundaries of ‘core gaming’, players might face rejection from peers in highly normative (and fiercely gatekept) online spaces (Chess and Paul, 2019). Current findings speak to this culture in one form and deviate from them in another. Yes, players expressed more of an interest in WDtCCtR than non-players, but they were also more likely to be apathetic to the topic the game centered on. This points at a discrepancy between the use of gaming to promote pro-social attitudes and behaviors. A deeper understanding is needed on the differences between the audience a game targets and the audience a game attracts in practice.

#### 4.2.2. Practical implications

Extrapolating from this study’s findings, we would advise that developers are more forthcoming about their games’ intent. There is a growing group of people who are interested in playing games that pertain to real-world topics and issues, or who at least are not shying away from games that advertise these issues outright. There is some leeway for those looking to market persuasive game experiences; texts can either emphasize their persuasive intent or present the game as any other entertainment game. However, aside from any ethical misgivings one might have about the latter tactic, the optimal outcome the current study points to is one in which games are presented in a congruous way as offering a deeper experience with ties to real-world issues.

In this study, we focused on games published on the independent gaming platform Itch.io. Though we did not look to include Itch.io’s regulars in the current sample, findings relating to attitudes toward gaming in general do tentatively point out that developers cannot rely on the tools these platforms offer alone when it comes to lending their project visibility to its target audience. First of all, it might be that the target audience diverges from gamers in the traditional sense. Itch.io’s player base clearly enjoys more diverse gaming experiences, but that does not mean they will be open toward persuasive games. Second, hosting a game on Itch.io places it among a fluid catalog of games that compete for attention of those browsing the platform. Unless people are specifically looking for keywords relating to the topic, there is little that separates “poem/game” experiences like WDtCCtR from thousands of entertainment games. Shifting focus from developers to platform holders, we would recommend focusing efforts to improve visibility of games by improving affordances for eWOM. Peer recommender systems are seen as more impactful than automated messages with personalized recommendations.

### 4.3. Limitations and recommendations

One of the major roadblocks to understanding the real-world impact persuasive games have is the tendency for validation research to center on captive audiences (Jacobs, 2021). Even though the current study did focus on the period between seeing a game and wanting to play it, it still employed a captive audience of study participants. Less than half of those players would select the persuasive game over a similar entertainment game, and less than a fifth of study participants ultimately sought out the game after the study ended without being asked to do so. If research into this aspect of persuasive gaming is continued, an ideal situation would be to perform online A/B-style testing directly on platforms. Different textual frames and other potential attractors could be explored for new visitors to a game’s page. Including more than just a simple log of what attractors make it more likely a game is actually played is challenging (on ethical and practical levels), but there is no doubt this would lead to more externally valid insights.

The approach of the current study centered the potential agency of developers and platform holders, rather than the experiences and motivations of players. The article can therefore only speak on how persuasive games might appeal to individuals who are already browsing the catalog of games on Itch.io and similar platforms. Judging from the finding that players who liked games in general were also more likely to play WDtCCtR, one could argue that more frequent users of these platforms would more readily select a persuasive game over an entertainment-centered one. However, evidence for this is not conclusive. To fill this gap, future research could involve more of the player’s journey. This includes understanding how gaming motivations (Holl et al., 2021) might relate to the selection process, but also to delve deeper into the seemingly growing sense players have that games gratify eudaimonic needs (Daneels et al., 2021a).

The current study’s effect sizes were small. While these might be indicative of a small real-world effect, the manipulations used here might have been a factor as well. To stay close to the short descriptions found on game pages on Itch.io, attraction texts were written succinctly, with just a few words and phrases differing between conditions. Moreover, only the texts were manipulated, with identical visuals being used for the entertainment and persuasive game. Stronger manipulations could be embedded in, for instance, video trailers for a game. An audiovisual presentation might hold viewers’ attention for longer, though of course care must be taken to avoid introducing new confounds.

Next, the stimuli were constructed around one specific persuasive game. The choice for WDtCCtR was made for various reasons (some of which are listed in the Materials and Methods section). Of course, the focus on a very specific example of one type of persuasive game limits generalizability in some respects. Further research should explore which topics do not suffer from reactance as a result of obtrusiveness and explicit persuasive intent, and which do. As an example of the latter, advergames’ emphasis on sales and brand attitudes might unilaterally benefit from obfuscation of persuasive intent.

Even though the current study is situated in the landscape of persuasive game validation research, it does not put us in a position to speak to the effects of WDtCCtR on players. Some of the complex findings relating player’s prior attitudes to interest and self-reported play behavior might translate to a validation setting. However, that in itself is not proof. These variables are clearly (at least partially) influenced by other factors that were not measured here. We would advise a qualitative investigation into the experiences people have when browsing for games on platforms like Itch.io. This would help us identify which factors might also play a role in this complex decision-making process.

Finally, the current study made use of a convenience sample. While the stimuli and measures were chosen and constructed around the population this study sampled from, the sample is not representative of this population. More research on representative subsets of the population is needed to understand the appeal of persuasive games for a wide audience of natural players, as well as to understand whether the mechanisms of frames and recommendations translate to the population as a whole. The influence of persuasive intent frames, specifically, might be highly dependent on digital skills and media literacy, factors which vary widely across groups of people. It is important to note, though, that persuasive games also tend to have defined target populations. Any follow-up research should find a balance between games’ intended players, issue relevance, and those populations who might benefit the most from engaging with this kind of persuasive communication.

## 5. Conclusion

The objective of this study was to chart the decision process potential players go through before they start playing a persuasive game called ‘Why did the chicken cross the road?’ that is available for free on gaming platforms. Two elements commonly found on these platforms were manipulated; the way a text can draw attention to the intent of the game to provide hedonic or eudaimonic gratifications or to persuade its players, and how such a game can come recommended by an automated system or by electronic word-of-mouth. Attitudes and behaviors were assessed at three points: interest was measured after reading attract texts and choosing for the game or an entertainment-focused alternative, intention to play was gaged after receiving a recommendation for the same game, and a final measurement asked respondents whether they played the game 1 week on from exposure.

The outcomes speak to different effects of frames and recommendations; frames seem to function as cues that are necessary but not sufficient in isolation, while game recommendations coming from a peer elicit greater play intention than automated versions. Interest and play intentions were strongest when frames and recommendations combined in a way that made the persuasive game’s intent explicit. One week after exposure, the manipulated variables no longer had an impact on whether or not respondents would seek out the game to actually play it, though a small minority of respondents still did so after their interest was piqued during the study. Respondents who had stronger affinity with games were more likely to try playing the game but were also more likely to be less willing to help in the real world.

One of the ways in which persuasive games could attract players is by earnestly stating their goals upfront. Respondents in conditions where intent was clearest did not react negatively to these stimuli and in fact were more likely to select the persuasive game over a comparable entertainment game. At the same time, the results speak to a discrepancy between the target audiences of persuasive games and avid players who might be roaming gaming platforms looking for content that entertains but that does not necessarily have to have a deeper meaning. Focusing on what happens before a play session is vital to understand the impact a persuasive game can have, and the current study allows for cautious optimism about the roles entertainment delivery platforms can have in bringing these games the audiences they are made for.

## Data availability statement

The raw data supporting the conclusions of this article are made available by the authors, without undue reservation. The dataset, syntax, and survey materials are available on OSF: https://doi.org/10.17605/OSF.IO/J7Y56.

## Ethics statement

The studies involving humans were approved by University of Twente Ethics Committee BMS, Domain Humanities & Social Sciences (request number 220404). The studies were conducted in accordance with the local legislation and institutional requirements. The participants provided their written informed consent to participate in this study.

## Author contributions

MG conceptualized, designed, performed, reported on, and finalized this study as part a master’s thesis. RJ was MG’s supervisor the throughout the process, and had overseen all aspects in a supporting role. MG defended the thesis. RJ wrote the current manuscript with elements from MG’s writing. RJ performed a new round of data analysis and replaced most of the results reporting in line with revised hypotheses. All authors contributed to the article and approved the submitted version.

## Conflict of interest

The authors declare that the research was conducted in the absence of any commercial or financial relationships that could be construed as a potential conflict of interest.

## Publisher’s note

All claims expressed in this article are solely those of the authors and do not necessarily represent those of their affiliated organizations, or those of the publisher, the editors and the reviewers. Any product that may be evaluated in this article, or claim that may be made by its manufacturer, is not guaranteed or endorsed by the publisher.

## Footnotes

^1^ https://doi.org/10.17605/OSF.IO/J7Y56

## References

[ref1] Ajzen I. (1991). The theory of planned behavior. Organ. Behav. Hum. Decis. Process. 50, 179–211. doi: 10.1016/0749-5978(91)90020-T

[ref2] Amelina D. Zhy Y. Q. (2016). Investigating effectiveness of source credibility elements on social commerce endorsement: the case of instagram in Indonesia. In Pacific Asia conference on information systems, PACIS 2016 – proceedings. Atlanta, Georgia, USA: AIS Electronic Library.

[ref3] Ashraf M. Sulaiman A. Jaafar N. I. (2018). System generated recommendation vs consumer generated recommendation: a differential effect on consumers beliefs and behavior in eCommerce transactions. Available at: http://aisel.aisnet.org/pacis2017/108

[ref4] Bacovsky P. (2021). Gaming alone: Videogaming and sociopolitical attitudes. New Media Soc. 23, 1133–1156. doi: 10.1177/1461444820910418

[ref5] Bourgonjon J. Valcke M. Soetaert R. Schellens T. (2010). Students’ perceptions about the use of video games in the classroom. Comput. Educ. 54, 1145–1156. doi: 10.1016/j.compedu.2009.10.022

[ref6] Brehm S. S. Brehm J. W. (1981). Psychological reactance: a theory of freedom and control. New York: Academic Press.

[ref7] Breuer J. Bente G. (2010). Why so serious? On the relation of serious games and learning. J. Comput. Game Cult. 4, 7–24. doi: 10.7557/23.6111

[ref8] Chess S. Paul C. A. (2019). The end of casual: long live casual. Games Cult. 14, 107–118. doi: 10.1177/1555412018786652

[ref9] Cheung M. Y. Luo C. Sia C.-L. Chen H. (2009). Credibility of electronic word-of-mouth: informational and normative determinants of on-line consumer recommendations. Int. J. Electron. Commer. 13, 9–38. doi: 10.2753/JEC1086-4415130402

[ref10] Crecente D. (2014). Gaming against violence: a grassroots approach to teen dating violence. Games Health J. 3, 198–201. doi: 10.1089/g4h.2014.0010, PMID: 26192368

[ref11] Daneels R. Bowman N. D. Possler D. Mekler E. D. (2021a). The ‘Eudaimonic experience’: a scoping review of the concept in digital games research. Media Commun. 9, 178–190. doi: 10.17645/mac.v9i2.3824

[ref12] Daneels R. Malliet S. Geerts L. Denayer N. Walrave M. Vandebosch H. (2021b). Greek warriors, Norse gods, and androids: how narratives and game mechanics shape eudaimonic game experiences. Media Commun. 9, 49–61. doi: 10.17645/mac.v9i1.3205

[ref13] DatJuanDesigner (2020). Why did the chicken cross the road?. Available at: https://juegos.itch.io/why-did-the-chicken-cross-the-road

[ref14] De Grove F. Courtois C. Van Looy J. (2015). How to be a gamer! Exploring personal and social indicators of gamer identity. J. Comput. Commun. 20, 346–361. doi: 10.1111/jcc4.12114

[ref15] Filieri R. Alguezaui S. McLeay F. (2015). Why do travelers trust TripAdvisor? Antecedents of trust towards consumer-generated media and its influence on recommendation adoption and word of mouth. Tour. Manag. 51, 174–185. doi: 10.1016/j.tourman.2015.05.007

[ref16] Friestad M. Wright P. (1994). The persuasion knowledge model: how people cope with persuasion attempts. J. Consum. Res. 21, 1–31. doi: 10.1086/209380

[ref17] Groen M. (2022). A game of persuasion: Investigating factors influencing player responses towards the presentation of a persuasive game. Available at: http://essay.utwente.nl/93242/.

[ref18] Hayes A. F. (2018). Introduction to mediation, moderation, and conditional process analysis: a regression-based approach. 2nd ed. New York, NY, USA: The Guilford Press.

[ref19] Holl E. Wagener G. L. Melzer A. (2021). “Motivation to play scale (MOPS): measuring gaming motivation with a comprehensive instrument” in 71st annual Internation communication association conference (Denver: Communication & mass media).

[ref20] Howe W. T. Livingston D. J. Lee S. K. (2019). Concerning gamer identity: an examination of individual factors associated with accepting the label of gamer. First Monday 24, 3–4. doi: 10.5210/fm.v24i3.9443

[ref21] Igartua J.-J. Wojcieszak M. Kim N. (2019). How the interplay of imagined contact and first-person narratives improves attitudes toward stigmatized immigrants: a conditional process model. Eur. J. Soc. Psychol. 49, 385–397. doi: 10.1002/ejsp.2509

[ref22] Jacobs R. S. (2017). Playing to win over: validating persuasive games, Erasmus University Rotterdam, Rotterdam.

[ref23] Jacobs R. S. (2021). Winning over the players: investigating the motivations to play and acceptance of serious games. Media Commun. 9, 28–38. doi: 10.17645/mac.v9i1.3308

[ref24] Jacobs R. S. Jansz J. (2021). “The present of persuasion: escalating research into persuasive game effects,” in Persuasive gaming in context, eds. Hera T. De la Jansz J. Raessens J. Schouten B. A. M. (Amsterdam, Netherlands: Amsterdam University Press).

[ref25] Kaufman G. Flanagan M. (2015). A psychologically “embedded” approach to designing games for prosocial causes. Cyberpsychology 9:3. doi: 10.5817/CP2015-3-5

[ref26] Kaufman G. Flanagan M. Seidman M. (2021). “Creating stealth game interventions for attitude and behavior change: an ‘embedded design’ model,” in Persuasive gaming in context, eds. Hera T. De la Jansz J. Raessens J. Schouten B. (Amsterdam University Press), Amsterdam.

[ref27] Kneer J. Jacobs R. S. Ferguson C. J. (2018). You could have just asked: the perception of motivations to play violent video games. Stud. Media Commun. 6:1. doi: 10.11114/smc.v6i2.3389

[ref28] Kudeshia C. Kumar A. (2017). Social eWOM: does it affect the brand attitude and purchase intention of brands? Manag. Res. Rev. 40, 310–330. doi: 10.1108/MRR-07-2015-0161

[ref29] Kümpel A. S. Unkel J. (2017). The effects of digital games on hedonic, eudaimonic and telic entertainment experiences. J. Gaming Virtual Worlds 9, 21–37. doi: 10.1386/jgvw.9.1.21_1

[ref30] Lin Z. (2014). An empirical investigation of user and system recommendations in e-commerce. Decis. Support. Syst. 68, 111–124. doi: 10.1016/j.dss.2014.10.003

[ref31] Luo C. Luo X. Schatzberg L. Sia C. L. (2013). Impact of informational factors on online recommendation credibility: the moderating role of source credibility. Decis. Support. Syst. 56, 92–102. doi: 10.1016/j.dss.2013.05.005

[ref32] Malliet S. Martens H. (2010). “Persuasive play: extending the elaboration likelihood model to a game based learning context,” in Interdisciplinary models and tools for serious games: Emerging concepts and future directions, ed. Eck R. Van (Hershey, PA, IGI Global).

[ref33] Mallinckrodt V. Mizerski D. (2007). The effects of playing an advergame on young children’s perceptions, preferences, and requests. J. Advert. 36, 87–100. doi: 10.2753/JOA0091-3367360206

[ref34] McConahay J. B. Hardee B. B. Batts V. (1981). Has racism declined in America? J. Confl. Resolut. 25, 563–579. doi: 10.1177/002200278102500401

[ref35] Metro Trains Melbourne (2016). Dumb Ways To Die - The PSA. Available at: https://www.dumbwaystodie.com/psa (Accessed August 08, 2023).

[ref36] Oliver M. B. Raney A. A. (2011). Entertainment as pleasurable and meaningful: identifying hedonic and Eudaimonic motivations for entertainment consumption. J. Commun. 61, 984–1004. doi: 10.1111/j.1460-2466.2011.01585.x

[ref37] Spears N. Singh S. N. (2004). Measuring attitude toward the brand and purchase intentions. J. Curr. Issues Res. Advert. 26, 53–66. doi: 10.1080/10641734.2004.10505164

[ref38] Staunton T. V. Alvaro E. M. Rosenberg B. D. (2022). A case for directives: strategies for enhancing clarity while mitigating reactance. Curr. Psychol. 41, 611–621. doi: 10.1007/s12144-019-00588-0

[ref39] Tsay-Vogel M. Krakowiak K. M. (2016). Effects of hedonic and Eudaimonic motivations on film enjoyment through moral disengagement. Commun. Res. Reports 33, 54–60. doi: 10.1080/08824096.2015.1117443

[ref40] Tutaj K. van Reijmersdal E. A. (2012). Effects of online advertising format and persuasion knowledge on audience reactions. J. Mark. Commun. 18, 5–18. doi: 10.1080/13527266.2011.620765

[ref41] van’t Riet J. Meeuwes A. C. van der Voorden L. Jansz J. (2018). Investigating the effects of a persuasive digital game on immersion, identification, and willingness to help. Basic Appl. Soc. Psych. 40, 180–194. doi: 10.1080/01973533.2018.1459301

[ref42] Vanwesenbeeck I. Walrave M. Ponnet K. (2017). Children and advergames: the role of product involvement, prior brand attitude, persuasion knowledge and game attitude in purchase intentions and changing attitudes. Int. J. Advert. 36, 520–541. doi: 10.1080/02650487.2016.1176637

[ref43] Wang Y. Hollett N. L. (2022). Cognitive, affective, and global attitude toward physical activity with different intensities. Int. J. Sport Exerc. Psychol. 20, 551–568. doi: 10.1080/1612197X.2020.1869803

